# Proposal for a definition for response to treatment, inactive disease and damage for JIA associated uveitis based on the validation of a uveitis related JIA outcome measures from the Multinational Interdisciplinary Working Group for Uveitis in Childhood (MIWGUC)

**DOI:** 10.1186/s12969-019-0345-2

**Published:** 2019-10-01

**Authors:** Ivan Foeldvari, Jens Klotsche, Gabriele Simonini, Clive Edelsten, Sheila T. Angeles-Han, Regitze Bangsgaard, Joke de Boer, Gabriele Brumm, Rosa Bou Torrent, Tamas Constantin, Cinzia DeLibero, Jesus Diaz, Valeria Maria Gerloni, Margarida Guedes, Arnd Heiligenhaus, Kaisu Kotaniemi, Sanna Leinonen, Kirsten Minden, Vasco Miranda, Elisabetta Miserocchi, Susan Nielsen, Martina Niewerth, Irene Pontikaki, Carmen Garcia de Vicuna, Carla Zilhao, Steven Yeh, Jordi Anton, Joan Calzada

**Affiliations:** 1 Head of the Hamburg Centre for Pediatric and Adolescence Rheumatology Centre for Treatment of Scleroderma and Uveitis in Childhood and Adolescence Teaching Unit of the Asklepios Campus of the Semmelweis Medical School, Budapest An der Schön Klinik Hamburg Eilbek Dehnhaide, 120 22081 Hamburg, Germany; 2 German Rheumatism Research Centre, 10117 Berlin, Germany; 3 Institute for Social Medicine, Epidemiology, and Health Economics, Charité Universitaetsmedizin Berlin, Berlin, Germany; 4 Rheumatology Unit- A. Meyer Children’s Hospital- NEUROFARBA Department, University of Florence, Florence, Italy; 5 Dept Rheumatology, Great Ormond Street Hospital, Great Ormond Street, London, UK; 6 Division of Rheumatology, Cincinnati Children’s Hospital Medical Center, 3333 Burnett Avenue, Cincinnati, OH 45229; Department of Pediatrics, University of Cincinnati, Cincinnati, OH USA; 7 Department of Ophthalmology, Copenhagen University Hospital Glostrup/Rigshospitalet, Copenhagen, Denmark; 8 UMC Utrecht, Utrecht, Netherlands; 9 Universitätsklinikum Hamburg-Eppendorf, Hamburg, Germany; 10 Pediatric Rheumatology Department, Hospital Sant Joan de Déu, Barcelona, Spain; 11 2nd Department of Pediatrics, Semmelweis University, Budapest, Hungary; 12 AOU Meyer, Florence, Italy; 13 Università di Milano - Istituto Gaetano Pini, Milan, Italy; 14 Pediatric Rheumatology Unit, Centro Hospitalar Universitário do Porto, Porto, Portugal; 15 St. Franziskus-Hospital Münster, Muenster, Germany; 16 Department of Ophthalmology, Helsinki University Hospital, Helsinki, Finland; 17 Charité – Universitätsmedizin Berlin, corporate member of Freie Universität Berlin, Humboldt-Universität zu Berlin, and Berlin Institute of Health, Department of Rheumatology and Clinical Immunology, Berlin, Germany; 18 Pediatric Ophthalmologist at the Centro Hospitalar Universitario do Porto, Teaching Unit of the Abel Salazar Institute of Biomedical Sciences, Largo do Prof. Abel Salazar, 4099-001 Porto, Portugal; 19 Ocular Immunology and Uveitis Service, Department of Ophthalmology, San Raffaele Scientific Institute, Via Olgettina 60, 20122 Milan, Italy; 20 Emory Clinic, Atlanta, USA; 21 Institut de Recerca Sant Joan de Déu, Barcelona, Spain; 22 Department of Surgery and Surgery Specializations. Universitat de Barcelona, Barcelona, Spain; 23 Ophtalmology Department, Hospital Sant Pau, Barcelona, Spain; 24 Ophtalmology Department, Hospital Sant Joan de Déu, Barcelona, Spain

**Keywords:** Anterior uveitis, Uveitis, Juvenile idiopathic arthritis, Response, Damage, Inactive disease, Outcome measures

## Abstract

**Background:**

JIA-associated uveitis (JIAU) is a serious, sight-threatening disease with significant long-term complications and risk of blindness, even with improved contemporary treatments. The MIWGUC was set up in order to propose specific JIAU activity and response items and to validate their applicability for clinical outcome studies.

**Methods:**

The group consists of 8 paediatric rheumatologists and 7 ophthalmologists. A consensus meeting took place on November 2015 in Barcelona (Spain) with the objective of validating the previously proposed measures. The validation process was based on the results of a prospective open, international, multi-centre, cohort study designed to validate the outcome measures proposed by the initial MIWGUC group meeting in 2012. The meeting used the same Delphi and nominal group technique as previously described in the first paper from the MIWGUC group (Arthritis Care Res 64:1365–72, 2012). Patients were included with a diagnosis of JIA, aged less than 18 years, and with active uveitis or an uveitis flare which required treatment with a disease-modifying anti-rheumatic drug. The proposed outcome measures for uveitis were collected by an ophthalmologist and for arthritis by a paediatric rheumatologist. Patient reported outcome measures were also measured.

**Results:**

A total of 82 patients were enrolled into the validation cohort. Fifty four percent (*n* = 44) had persistent oligoarthritis followed by rheumatoid factor negative polyarthritis (*n* = 15, 18%). The mean uveitis disease duration was 3.3 years (SD 3.0). Bilateral eye involvement was reported in 65 (79.3%) patients.

The main findings are that the most significant changes, from baseline to 6 months, are found in the AC activity measures of cells and flare. These measures correlate with the presence of pre-existing structural complications and this has implications for the reporting of trials using a single measure as a primary outcome. We also found that visual analogue scales of disease activity showed significant change when reported by the ophthalmologist, rheumatologist and families.

The measures formed three relatively distinct groups. The first group of measures comprised uveitis activity, ocular damage and the ophthalmologists’ VAS. The second comprised patient reported outcomes including disruption to school attendance. The third group consisted of the rheumatologists’ VAS and the joint score.

**Conclusions:**

We propose distinctive and clinically significant measures of disease activity, severity and damage for JIAU. This effort is the initial step for developing a comprehensive outcome measures for JIAU, which incorporates the perspectives of rheumatologists, ophthalmologists, patients and families.

**Electronic supplementary material:**

The online version of this article (10.1186/s12969-019-0345-2) contains supplementary material, which is available to authorized users.

## Introduction

Juvenile Idiopathic Arthritis (JIA) is the most common extra-ocular disease associated with childhood chronic anterior uveitis and has a well-developed body of internationally agreed case definitions, outcome measures and disease specific quality of life metrics that support clinical trials and health economic assessments. JIA-associated uveitis (JIAU) is a serious, sight-threatening disease with significant long-term complications and risk of blindness even with improved contemporary treatments [2, 3]. In contrast to JIA, international consensus on definitions and management [4, 5], or randomised trials [6], have only recently been developed in JIAU.

Recent JIA cohorts confirm an annual incidence of uveitis of 2–4% in the first years after the onset of arthritis [5, 7] and an estimated cumulative incidence of 10–20%. JIAU is a rare cause of uveitis in the general population and comprises only 1–2% of all cases of uveitis. Even in tertiary uveitis care settings JIAU comprises only 4 to 33% of cases [7–11]. The rarity of JIAU and lack of similarity to more common types of uveitis has contributed to the scarcity of agreed terminology and outcome measures Although treatments may simultaneously improve control of both arthritis and uveitis there is low correlation between the activity and damage from the two conditions. Without common measures of treatment effects on both arthritis and uveitis, trials have been conducted separately on each organ system, which is an inefficient use of resources. Understanding the relationship between treatment effects on both arthritis and uveitis in JIA may improve patient outcomes and shared care protocols.

There are now several agreed criteria for judging the quality of data used in observational studies and trials. Current evidence for the application of outcome measures comes from case series, retrospective studies and heterogeneous populations. Most of the measures used in JIAU trials do not fulfil the OMERACT (Outcome Measures in Rheumatology) criteria or COMET (Core Outcome Measures in Effectiveness Trials) guidelines [13–15]. Published outcomes of both trials and observational studies remain varied and studies are rarely designed to replicate, or build on, previous findings (Mastrangelo et al., submitted review of outcome measures in this journal) and most published studies, do not use child-appropriate outcome measures [12].

The Multinational Interdisciplinary Working Group for Uveitis in Childhood (MIWGUC) group was set up in order to address these deficiencies in the quality of assessment and trial design in patients with JIAU [1]. The sharing of data and results is difficult without standardisation of methodology. Even the most important measure shared as an outcome between doctors patients and health providers, visual acuity, has a large number of methods of measurement, which vary by age group, and whose replicability is highly patient- and operator--dependent.

The evaluation and management of children with JIAU requires close collaboration between ophthalmologists and paediatric rheumatologists, and measures endorsed by both subspecialties is necessary.

The Standardization of Uveitis Nomenclature (SUN) Working Group reported a standardized nomenclature of uveitis, inflammation grading, and outcome measures [16], providing a validated instrument for comparing patient data, in routine clinical practice as well as trials. The SUN criteria are generic and JIAU requires disease-specific criteria due to its childhood onset, the lack of an equivalent pattern of uveitis in adults and the frequent comorbidity of active arthritis. The MIWGUC group has prioritized reviewing the SUN criteria to select specific JIAU activity and response items and validate their applicability for clinical outcome studies.

The COMET programme emphasises that developing useful core outcome measures requires both evidence from carefully controlled short-term randomised trials as well as effectiveness-trials that more closely resemble routine clinical practice in both the range of severity of disease and the variety of previous treatments.

In this study the MIWGUC group reports the results of a 6 months observational study of JIAU patients thought by their practitioner to require the introduction of a new DMARD. The purpose was to evaluate those measures that were most sensitive to change following clinically indicated escalation of treatment and to determine whether a variety of clinical measures and physician- and patient-reported evaluations of response and morbidity provided additional, or unique, perspectives on the changes in disease following treatment escalation.

A consensus statement was developed suggesting the most relevant domains for future studies. The range of preferred items provides a framework that might be used to test more disease-specific patient-related outcome measures and novel methods of clinical measurements. In this way we hope to improve the link between short-term changes appropriate for study in efficacy trials and the resultant lifelong morbidities of most relevance to patients and those funding health interventions.

The wide range of clinical management of JIAU remains a significant concern for treating rheumatologists and ophthalmologists and enlarging an evidence-based consensus between specialists as well as between specialists and patients is essential to improve outcomes [3, 18].

## Methods

### Consensus process

The MIWGUC group consists of 8 paediatric rheumatologists and 7 ophthalmologists who have a special interest in JIAU. A meeting took place on the 19th to 21st of November 2015 in Barcelona (Spain) with the objective of validating the previously proposed outcome measures [1]. Firstly, the results from the validation cohort that are reported and discussed in this paper were presented. The participants then selected the relevant items to assess uveitis activity, uveitis damage and responsiveness to treatment over 6 months using a nominal group technique [19] during the consensus development process. No patient or patients’ representative participated in the process.

### Patient selection

We conducted an open, international, multi-centre, prospective cohort study to prospectively validate the outcome measures proposed by the initial MIWGUC group meeting in 2009 [1]. Patients (i) with a diagnosis of JIA according to the ILAR (International League of Associations for Rheumatology) classification [20], (ii) aged less than 18 years, and (iii) presence of active uveitis at least in one eye (≥ 1+ anterior chamber (AC) cells grade according the SUN criteria) or uveitis flare which required either the initiation of treatment with a conventional synthetic disease-modifying anti-rheumatic drug (csDMARD) or with a biologic DMARD (bDMARD) as indicated by the treating physician for the uveitis. The patients did not to have to be naive to DMARD treatment.

The patients were assessed by a paediatric rheumatologist and an ophthalmologist at study enrolment, at 3 months, and 6 months follow-up. Additionally, patient-reported outcome measures were collected at each assessment.

### Measures

#### Ophthalmology outcomes

The ophthalmologist documented clinical characteristics of uveitis, onset date, the number of affected eyes and the anatomical classification of uveitis (anterior, intermediate, posterior, panuveitis). Uveitis activity was assessed by slit lamp examination of the anterior segment and fundoscopy. The following activity measures were collected for each affected eye: the total number of AC (anterior chamber) cells, AC cell grade before and after pupil dilatation (grade 0 (< 1 cells), grade 0.5+ (1–5 cells), 1+ (6–15 cells), 2+ (16–25 cells), 3+ (26–50 cells), 4+ (> 50 cells)), AC flare grade according to the SUN and MIWGUC criteria [1, 16], and visual acuity. The laser flare photometry values were added when available. The MIWGUC criteria for flare have less response categories (no flare, moderate, intense, not possible) compared to the SUN criteria (no flare, faint, moderate, marked, intense, not possible). Visual acuity was documented by a logMAR (logarithm of the minimum angle of resolution) chart. Structural eye complications and current topical eye treatment were also entered by the ophthalmologist. The ophthalmologist assessed the overall uveitis activity on a visual analogue scale (VAS, range 0 to 100 mm, 0 = inactive disease).

#### Rheumatology outcomes

The paediatric rheumatologist reported clinical JIA parameters: age at onset, disease duration, the number of joints with active arthritis, treatment with csDMARDs and bDMARDs, concomitant treatment with systemic glucocorticoids and non-steroidal anti-inflammatory drugs (NSAIDs) as well as overall disease activity on a VAS (range 0 to 100 mm, 0 = inactive disease).

#### Patient-related outcomes

The parents reported the days of hospitalization due to uveitis, lost days in kindergarten or school and restrictions in daily activities due to uveitis for the last 6 months. The assessment of overall well-being was assessed by families on a VAS (range 0 to 100 mm, 0 = best possible value). Functional complications were assessed by the Childhood Health Assessment Questionnaire (CHAQ, range 0–3, 0 = no functional limitations [21]) and quality of life by the Paediatric Quality of Life Inventory (PedsQL, range 0–100, 100 = best possible quality of life [22]). A summary of the measures is shown in Table 1.

**Table 1:** Summary of tested outcome measures in the prospective study in patients with JIAU. Summary of tested outcome measures in the international, multi-centre, prospective, and uncontrolled cohort study as proposed by Heiligenhaus et al. [14]

Paediatric Rheumatologist • Physician ‘ s global assessment of disease activity on a visual analogue scale , 0-100 • Number of joints with active arthritis	Ophthalmologist • Total number of AC cells • AC cell grade before and after dilatation • ACflare grade according to the SUN criteria and MIWGUC criteria • Ophthalmologists global assessment of uveitis activity on a visual analogue scale, 0-100 • Visual acuity, LogMAR • Presence of structural complications – Ocular hypotony (IOP ≤ 6 mmHg) – Ocular hypertension – Posterior synechiae formation – Glaucoma – Cataract – Band keratopathy in the cornea – Optic disc edema – Macular edema – Epiretinal membrane formation – Vitreous haze	Patient‘s reported outcome • Overall well-being on a visual analogue scale, 0-100 • Functional ability by C-HAQ • Quality of life by PedsQL • Days of hospitalizations due to uveitis • Lost days in kindergarden or school due to uveitis • Days with restrictions in daily life due to uveitis

### Statistics

Parameters that were related to JIA disease characteristics and the patient reported outcomes were reported at the patient level, whereas parameters that were related to uveitis were reported at the eye level. The sensitivity to change within the 6 months observation period was investigated by linear mixed models. The change in each parameter was estimated between baseline to 3-months follow-up, baseline to 6-months follow-up and 3-months to 6-months follow-up within a linear mixed model. The number of affected eyes was additionally included as cluster variable in the analyses on the eye level. Preliminary analyses suggested that there was no selective loss to follow-up in our study. Linear mixed models result in reliable effect estimates in the presence of missing data and non-selective drop-out [23]. The association between all the considered parameters were studied by linear mixed models including the eye level as a cluster variable within the patient level using the patient identification number. All statistical analyses were conducted with STATA 12.1 (StataCorp. 2011. *Stata Statistical Software: Release 12*. College Station, TX: StataCorp LP). *P* values less than 0.05 were considered significant.

## Results

### Patient characteristics

A total of 82 patients were recruited in 10 study centres between January 2013 and June 2015. Sixty patients (73%) completed follow up at 6-months by the paediatric rheumatologist and the ophthalmologist. There was no statistically significant difference in socio-demographic and clinical characteristics at baseline between patients with and without 6 months follow-up except for the age at JIA onset (3.4 years, SD 2.6 versus 5.5 years, SD 4.7). The patient reported outcome measures were only available for 51 patients (62% of 82) at baseline and for 32 patients (39%) at the 6 months follow-up due to administrative reasons. The baseline characteristics of the study sample are shown in Tables 2 and 3.

**Table 2:** Sociodemographic and clinical characteristics of enrolled patients. Sociodemographic and clinical characteristics of the patients at study enrolment

	Total sample *N* = 82
Female sex	62 (75.6%)
ANA positive	60 (73.1%)
*HLA-B27* positive	8 (9.8%)
Rheumatoid factor positive	7 (8.5%)
Clinical JIA Characteristics (*n* = 82)	
Oligoarthritis, persistent	44 (53.7%)
Oligoarthritis, extended	9 (11.0%)
RF- Polyarthritis	15 (18.3%)
Psoriatic arthritis	1 (1.2%)
Enthesitis-related arthritis	4 (4.9%)
Undifferentiated JIA	4 (4.9%)
Unknown*	5 (6.1%)
JIA disease duration, years, mean (SD)	4.8 (3.8)
Age at disease onset in years, mean (SD)	3.8 (3.2)
Physician’s global, VAS, mean (SD) (range 0–100)	34.0 (28.7)
Patient reported outcomes (*n* = 51)	
Number of days in hospital due to uveitis in the last 6 months	12 (23.5%)
Missed days in kindergarten/ school due to uveitis in the last 6 months	27 (52.9%)
Number of days with restrictions in daily life due to uveitis in the last 6 months	12 (23.5%)
Patients assessment of overall well-being, VAS, mean (SD) (range 0–100)	34.1 (29.7)
C-HAQ, mean (SD) (range 0–3)	0.81 (1.04)
PedsQL, mean (SD) (range 0–100)	78.7 (21.5)
Clinical Uveitis Characteristics (*n* = 82)	
Uveitis disease duration, years, mean (SD)	3.3 (3.0)
Age at uveitis onset in years, mean (SD)	5.1 (2.9)
Number of affected eyes	
unilateral	17 (20.7%)
bilateral	65 (79.3%)
Physician’s global, VAS, mean (SD)	44.3 (34.1)
Number of patients with at least one active eye (AC cell grade > 0)	82 (100.0%)

*AC* anterior chamber, *ANA* anti-nuclear antibody, *CHAQ* Childhood Health Assessment Questionnaire, *HLA* human leukocyte antigen, *PedsQL* Paediatric Quality of Life Inventory, *RF* rheumatoid factor, *SD* standard deviation, *VAS* visual analogue scale range 0 to 100.* The category unknown includes patients with JIA for which the category was not reported by the paediatric rheumatologist.

**Table 3:** Clinical characteristics of eyes with uveitis. Clinical characteristics of uveitis on eye level at enrolment

	*N* = 147 affected eyes
AC cell grade (number of cells per hpf) according to SUN examined after dilatation	
0 [< 1]	30 (20.4%)
0.5+ [1–5]	16 (10.9%)
1+ [6–15]	53 (36.1%)
2+ [16–25]	26 (17.7%)
3+ [26–50]	9 (5.1%)
4+ [> 50]	2 (1.4%)
Missing	11 (7.5%)
Visual acuity, logMAR, mean (SD)	0.48 (0.51)
logMAR ≥0.1 (≤ 20/50)	79 (66.4%)
logMAR ≥1 (≤ 20/200)	5 (4.2%)
Any structural complication	81 (56.3%)
Ocular hypotony	3 (2.1%)
Ocular hypertension	6 (4.3%)
Posterior synechiae	60 (43.2%)
Glaucoma	6 (4.4%)
Cataract	28 (19.9%)
Band keratopathy	31 (21.5%)
Optic disc edema	10 (10.9%)
Macular edema	14 (21.5%)
Epiretinal membrane formation	7 (11.3%)
Vitreous haze, mean (SD)	1.37 (0.92)
Other complications	12 (12.8%)
Concomitant ocular uveitis treatment	
Previous subtenon / intraocular steroid injections	2 (1.4%)
Topical corticosteroid medication	76 (51.7%)
Glaucoma medication	10 (6.8%)

*AC* anterior chamber, *LogMAR* logarithm of the minimum angle of resolution, *SD* standard deviation, *VAS* visual analogue scale

The mean age for the 82 patients was 8.9 years (SD 3.7) at study inclusion. Approximately three out of four patients were female and 77 (94%) were Caucasian. More than half of the patients (*n* = 44, 54%) had persistent oligoarthritis followed by rheumatoid factor (RF) negative polyarthritis (*n* = 15, 18%). Sixty (73%) patients were anti-nuclear antibody (ANA) positive. The mean JIA and uveitis disease duration was 4.8 (SD 3.8) and 3.3 years (SD 3.0) years at baseline, respectively. Bilateral eye involvement was reported for 65 (79.3%) of the patients at baseline resulting in 147 eyes with uveitis. All patients had an active uveitis (AC cell grade above 0) in at least one eye at baseline.

All patients were treated with a DMARDs at baseline, 85.3% with a csDMARD (methotrexate 75.5%, azathioprine 14.7%) and 67.7 with a bDMARD (adalimumab 35.3%, infliximab 20.6%, tocilizumab 11.8%). 32.4% were treated with csDMARD only, 14.7% with a bDMARD only and 52.9% with a combination of a csDMARD and bDMARD.

#### Sensitivity to change between baseline and 6-months follow-up

Sensitivity to change between baseline and the 6-months follow-up was assessed to identify the parameters that may change after starting treatment. The detailed results were reported in Table 4.

**Table 4:** Change in parameters between baseline and 6-months follow-up. Change in parameters that are associated with JIA (paediatric rheumatologist) and uveitis (ophthalmologist) and patient reported outcome measures from baseline to 6-months follow-up (bold highlighted beta/ OR were statistically significant)

	Baseline	3-months follow-up	6-months follow-up	Change between Baseline and 3-months follow-up	Change between Baseline and 6-months follow-up	Change between 3-months and 6-months follow-up	Effect Size Baseline to 6-months follow-up
	n; mean (sd), median	n; mean (sd), median	n; mean (sd), median	beta/ OR *p* value 95% CI	beta/ OR *p* value 95% CI	beta/ OR *p* value 95% CI	
	n(%)^a^	n(%)^a^	n(%)^a^				
	Analyses on patient level						
	*n* = 82	*n* = 77	*n* = 64				
Pediatric rheumatologist							
Physician’s global about JIA disease activity, VAS, 0–100	71; 34.0 (28.7); 30	64; 17.0 (22.6); 10	57; 13.4 (22.4); 5	**−17.7 < 0.001–23.29; −12.14**	**−20.2 < 0.001–26.09; −14.41**	−2.5 0.401–8.46; 3.38	0.66
Number of joints with active arthritis	83; 0.6 (1.5); 0	77; 0.4 (1.5); 0	64; 0.9 (3.8); 0	−0.1 0.636–0.77; 0.47	0.3 0.313–0.32; 1.00	0.5 0.152–0.18; 1.15	0.10
Patients reported outcomes							
Overall well-being; VAS; 0–100	47; 34.1 (29.7); 26	43; 23.5 (20.3); 20	30; 23.2 (22.3); 12	**−10.5 0.003–17.48; −3.62**	**−13.1 0.001–20.93; −5.27**	− 2.6 0.524–10.39; 5.29	0.41
C-HAQ, 0–3	35; 0.81 (1.04); 0.5	27; 0.69 (0.88); 0.5	18; 0.52 (0.59); 0.44	− 0.2 0.056–0.45; 0.01	**− 0.3 0.014–0.61; − 0.07**	−0.1 0.4–0.39; 0.16	0.13
PedsQL, 0–100	52; 78.7 (21.5); 87.8	40; 81.7 (17.9); 86.9	26; 85.5 (13.5); 89.7	3.8 0.088–0.57; 8.21	**7.2 0.006 2.08; 12.30**	3.4 0.201–1.80; 8.54	0.15
Number of days in hospital due to uveitis	40; 1.9 (6.0); 0	32; 0.7 (2.7); 0	19; 1.1 (4.2); 0	−1.2 0.274–3.33; 0.94	−0.7 0.561–3.26; 1.77	0.4 0.736–2.16; 3.06	0.25
Missed days in kindergarden/ school due to uveitis	51; 4.8 (6.7); 1	47; 3.2 (9.2); 0	32; 0.5 (1.6); 0	−1.7 0.211–4.30; 0.95	**−4.3 0.004–7.25; − 1.34**	−2.6 0.085–5.61; 0.37	0.20
Number of days with restrictions in daily life due to uveitis	39; 7.4 (16.9); 0	32; 1.7 (4.8); 0	19; 5.0 (10.6); 0	**−5.9 0.037–11.40; − 0.36**	−2.7 0.417–9.25; 3.83	3.2 0.355–3.55; 9.90	0.44
	Analyses on eye level						
	*n* = 147	*n* = 132	*n* = 103				
Ophthalmologist							
Physician’s global about disease activity in the eyes, VAS, 0–100	113; 45.2 (33.9); 50	111; 30.6 (33.5); 20	87; 25.2 (32.3); 14	**−15.0 < 0.001–19.13; − 10.83**	**−20.0 < 0.001–24.57; − 15.44**	**− 5.0 0.029–9.55; − 0.51**	0.43
Total number of AC cells	46; 7.4 (6.4); 7.5	28; 1.4 (3.2); 0	33; 1.9 (4.8); 0	**−4.9 < 0.001–6.99; − 2.79**	**− 5.0 < 0.001–7.03; − 2.91**	−0.1 0.942–2.29; 2.12	1.10
Visual acuity, logMAR	119; 0.48 (0.51); 0.3	101; 0.39 (0.50); 0.1	86; 0.42 (0.52); 0.11	**−0.1 0.015–0.11; − 0.01**	−0.1 0.062–0.10; 0.00	0.0 0.727–0.04; 0.06	0.18
AC cell grade	136; 2.81 (1.25); 3	116; 1.81 (1.20); 1	93; 1.39 (0.82); 1	**−1.0 < 0.001–1.26; − 0.80**	**−1.5 < 0.001–1.72; − 1.22**	**−0.4 0.001–0.69; − 0.19**	0.81
AC flare grade (no flare versus flare), SUN	99 (70.2%)	49 (38.6%)	24 (25.3%)	**0.1 < 0.001 0.03; 0.21**	**0.0 < 0.001 0.01; 0.08**	**0.3 0.006 0.12; 0.71**	0.67
AC flare grade (no flare versus flare), MIWGUC	53 (61.6%)	31 (37.4%)	17 (25.8%)	**0.1 < 0.001 0.03; 0.34**	**0.0 < 0.001 0.01; 0.14**	**0.3 0.032 0.09; 0.90**	0.50
Any structural complications	81 (56.3%)	73 (56.2%)	64 (62.1%)	0.9 0.867 0.27; 2.99	1.1 0.904 0.28; 4.28	1.2 0.79 0.30; 4.77	0.01
Ocular hypotony	3 (2.1%)	3 (2.4%)	0 (0.0%)	^b^	^b^	^b^	0.02
Ocular hypertension	6 (4.3%)	8 (6.5%)	4 (4.1%)	6.2 0.235 0.31; 124.32	0.2 0.321 0.01; 4.05	0.0 0.109 0.00; 2.06	0.09
Posterior synechiae	60 (43.2%)	60 (46.2%)	53 (52.0%)	5.7 0.23 0.33; 96.67	4.7 0.324 0.21; 104.42	0.8 0.903 0.05; 15.33	0.06
Glaucoma	6 (4.4%)	6 (5.1%)	5 (5.4%)	^b^	^b^	^b^	0.03
Cataract	28 (19.9%)	23 (18.3%)	24 (23.5%)	^b^	^b^	^b^	0.04
Band keratopathy	31 (21.5%)	31 (24.6%)	27 (26.7%)	25.6 0.067 0.80; 826.75	14.8 0.132 0.44; 493.90	0.6 0.716 0.03; 11.13	0.07
Optic disc edema	10 (10.9%)	6 (7.0%)	3 (4.8%)	^b^	^b^	^b^	0.11
Macular edema (Funduscopy)	9 (7.0%)	4 (5.0%)	2 (2.9%)	0.3 0.288 0.04; 2.68	0.2 0.195 0.02; 2.14	0.7 0.811 0.06; 9.42	0.07
Macular edema (OCT)	14 (21.5%)	4 (12.5%)	2 (6.1%)	^b^	^b^	^b^	0.08
Epiretinal membrane formation (Funduscopy)	7 (5.5%)	7 (8.5%)	4 (5.9%)	^b^	^b^	^b^	0.12
Epiretinal membrane formation (OCT)	7 (11.3%)	7 (17.5%)	5 (14.3%)	^b^	^b^	^b^	0.11
Vitreous haze (yes/no)	15 (16.3%)	15 (18.3%)	13 (19.1%)	1.6 0.518 0.38; 6.97	1.5 0.632 0.31; 6.75	0.9 0.89 0.20; 4.11	0.06
Other complications	12 (12.8%)	2 (2.4%)	0 (0.0%)	^b^	^b^	^b^	0.10

*beta* regression coefficient for continuously distributed variables, *CI* confidence interval, *n* number of patients with valid assessment in the reported parameter, *OR* Odds ratio for categorical variables, *sd* standard deviation

^a^percentages refer to the number of patients or eyes with valid assessments in the considered parameter

^b^not estimable due to the low number of complications

### Ophthalmologic outcomes

The global assessment of uveitis activity on VAS by the ophthalmologist, the number of AC cells and the AC cell grade improved during the 6-months follow-up. There were significant improvements in AC flare grade at the 6-months follow-up. Visual acuity did not significantly improve (LogMAR baseline: mean = 0.48 (SD 0.51), LogMAR 3-months follow-up: mean = 0.42 (SD 0.52), beta = − 0.05, 95%CI: − 0.10; 0.001) during the study period. The number of eyes with impaired vision (≤20/50) improved slightly over the 6 months (79 (66.4%) of 119 eyes at baseline, 52 (60.5%) of 86 eyes at 6-months follow-up, *p* = 0.447). The decrease was more pronounced in eyes without glaucoma or macular edema, but also not statistically significant (64 (64.0%) of 100 eyes at baseline, 43 (56.6%) of 76 eyes at 6-months follow-up).

**Fig. 1:**
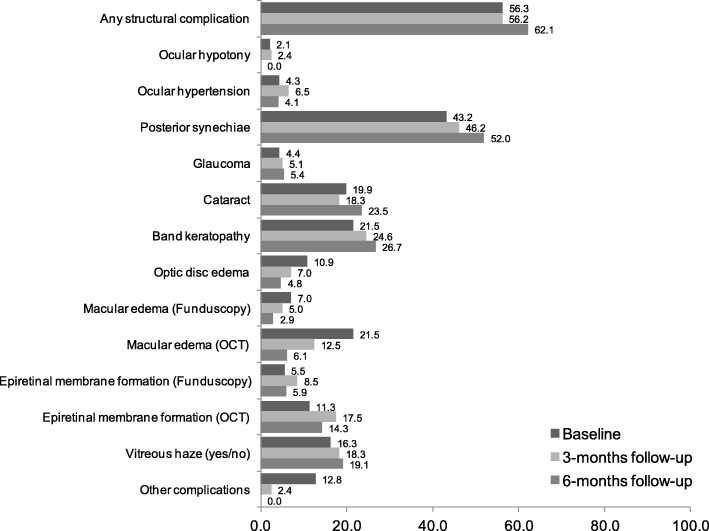
Proportion of eyes with structural complication. Proportion of eyes with any structural complication and specific structural complications (baseline *n* = 147 eyes, 3-months follow-up *n* = 132 eyes, 6-months follow-up *n* = 103 eyes)

Structural complications were found in 81 eyes (56.3% of 144 reported) at baseline and for 64 eyes (62.1% of 103) at 6-months follow-up (Fig. 1). There was no significant change in the frequency of eyes with structural complications overall or for any single complication over the 6 months.

The change in specific complications over time was investigated in 60 patients (103 eyes) with available 6-months follow-up data. Patients who were not included in the analyses (22 patients, 44 eyes) did not significantly differ in respect to uveitis disease characteristics and activity from the entire sample. However, the excluded patients showed a lower rate of structural complications at baseline (*n* = 17, 39%). In patients who could be investigated for 6-months, 64 (62.8%) eyes had at least one structural complication at baseline which persisted at the 6 months follow up (Fig. 1 and Table 5). New posterior synechiae formation was reported in 3 eyes (2.9%), a new cataract in 6 eyes (5.8%) and new vitreous haze in 5 eyes (4.9%) at 6- months follow-up. Optic disc edema (*n* = 7, 6.8%), ocular hypotony (IOP ≤ 6 mmHg, *n* = 2, 2.9%) and ocular hypertension (IOP (Intraocular pressure)) ≥ 21 mmHg, *n* = 2, 2.9%) reported at baseline was no longer reported at 6- months follow-up. More detailed information about the change in structural complications was reported in Table 5.

**Table 5:** Change in structural complication between baseline and 6-months follow-up. Change in structural complication within the 6 months follow-up

	Baseline		3-months follow-up						6-months follow-up					
			Changes in structural complications between baseline and 3-months follow-up						Changes in structural complications between baseline and 6-months follow-up					
	(*n* = 103 eyes)		(*n* = 103 eyes)						(*n* = 103 eyes)					
	total		total		newly reported		no longer reported		total		newly reported		no longer reported	
	N	%	N	%	N	%	N	%	N	%	N	%	N	%
Any structural complication	64	62.8	61	61.6					64	62.1				
Ocular hypotony (IOP ≤ 6 mmHg) during the last 3 months	3	2.9	3	2.9	1	1.0	1	1.0	0	0.0	0	0.0	3	2.9
Ocular hypertension (IOP ≥ 21 mmHg) during the last 3 months	6	5.8	6	5.8	2	1.9	2	1.9	4	3.9	1	1.0	3	2.9
Posterior synechiae formation	50	48.5	51	49.5	1	1.0	0	0.0	53	51.5	3	2.9	0	0.0
Glaucoma	4	3.9	3	2.9	0	0.0	1	1.0	5	4.9	1	1.0	0	0.0
Cataract	18	17.5	17	16.5	2	1.9	3	2.9	24	23.3	6	5.8	0	0.0
Band keratopathy in the cornea	25	24.3	25	24.3	2	1.9	2	1.9	26	22.9	2	1.9	0	0.0
Optic disc edema	10	9.7	6	5.8	0	0.0	4	3.9	3	2.9	0	0.0	7	6.8
Macular edema	10	9.7	5	4.9	2	1.9	7	6.8	3	2.9	1	1.0	8	7.8
Epiretinal membrane formation	5	4.9	5	4.9	0	0.0	0	0.0	5	4.9	0	0.0	0	0.0
Vitreous haze	14	13.6	13	12.6	4	3.9	5	4.9	13	12.6	5	4.9	6	5.8

### Rheumatological outcomes

The global assessments of JIA disease activity by the paediatric rheumatologist significantly improved. This improvement could be observed mainly in the first 3 months (beta = − 17.7, 95%CI: − 23.3; − 12.1 for change between baseline and 3-months follow-up; beta = − 2.5, 95%CI: − 8.5; 3.4 for change between 3-months and 6-months follow-up). A total of 21 patients (25.6%) had at least one joint with active arthritis at baseline. The number of joints with active arthritis and the proportion of patients with at least one active joint did not significantly change.

### Patient reported outcomes

Patient reports of overall well-being improved significantly from baseline levels. Functional ability (C-HAQ, beta = −.34, 95%CI: − 0.61; − 0.07) and quality of life (PedsQL, beta = 7.2, 95%CI: 2.1; 12.3) improved steadily over the 6 months. The number of missed days in kindergarten or school (beta = − 4.3, 95%CI: − 7.3; − 1.3) as well as the number of days with restrictions in daily life due to the uveitis (beta = − 5.9, 95%CI: − 11.4; − 0.4) significantly decreased at 6 and 3 months, respectively. There was no change in the number of hospital visits.

#### Association of uveitis anterior chamber activity and structural complications

The presence of structural complications was associated with AC cell grade and AC flare grade (Table 6). The strength of association between AC cell and flare grade was mediated by the presence of structural complications. At baseline, among eyes without any structural complications the AC flare grade was “no flare” in 81% of the eyes with an AC cell grade of zero and in 29% of the eyes with an AC cell grade greater or equal to one. In contrast, among eyes with at least one structural complication the AC flare grade was “no flare” in 46% of the eyes with an AC cell grade of zero and in 13% in eyes with an AC cell grade greater than zero. Similar results were found if posterior synechiae were the only complication considered.

**Table 6:** Association of AC cell grade and AC flare grade. Association of AC cell grade and AC flare grade in eyes with and without any structural complication

	All eyes				Eyes with at least one structural complications				Eyes with no structural complications			
	AC cell grade				AC cell grade				AC cell grade			
	0	0.5+	1+	2+/ 3+/ 4+	0	0.5+	1+	2+/ 3+/ 4+	0	0.5+	1+	2+/ 3+/ 4+
Baseline												
AC flare grade: no flare	20 (66.7%)	5 (31.3%)	9 (17.0%)	7 (18.9%)	6 (46.2%)	2 (25.0%)	3 (10.3%)	3 (12.0%)	13 (81.3%)	3 (37.5%)	6 (25.0%)	4 (33.3%)
AC flare grade: any flare	10 (33.3%)	11 (68.8%)	44 (83.0%)	30 (81.1%)	7 (53.8%)	6 (75.0%)	26 (89.7%)	22 (88.0%)	3 (18.8%)	5 (62.5%)	18 (75.0%)	8 (66.7%)
3-months follow-up												
AC flare grade: no flare	56 (83.6%)	6 (30.0%)	2 (14.3%)	2 (16.7%)	23 (82.1%)	4 (26.7%)	2 (18.2%)	2 (22.2%)	33 (84.6%)	2 (40.0%)	0 (0.0%)	0 (0.0%)
AC flare grade: any flare	11 (16.4%)	14 (70.0%)	12 (85.7%)	10 (83.3%)	5 (17.9%)	11 (73.3%)	9 (81.8%)	7 (77.8%)	6 (15.4%)	3 (60.0%)	3 (100.0%)	3 (100.0%)
6-months follow-up												
AC flare grade: no flare	60 (87.0%)	1 (12.5%)	2 (33.3%)	0 (0.0%)	33 (82.5%)	1 (16.7%)	0 (0.0%)	0 (0.0%)	27 (93.1%)	0 (0.0%)	2 (66.7%)	0 (0.0%)
AC flare grade: any flare	9 (13.0%)	7 (87.5%)	4 (66.7%)	4 (100.0%)	7 (17.5%)	5 (83.3%)	3 (100.0%)	4 (100.0%)	2 (6.9%)	2 (100.0%)	1 (33.3%)	0 (0.0%)

All patients had active uveitis at baseline in at least one eye. Among all eyes that are affected with uveitis were 20 eyes inactive and had no flare at baseline

#### Association of subjective meassures and quality of life and functional assessments

**Fig. 2:**
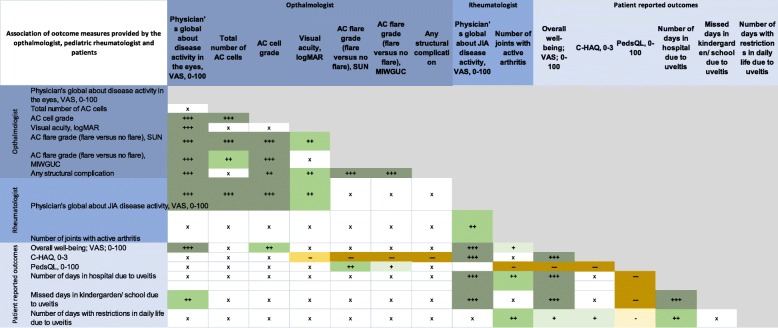
Summary of association of parameters that were assessed by the ophthalmologist, paediatric rheumatologist and patients. Association of parameters that were assessed by the *ophthalmologist, paediatric rheumatologist and patients* (‘+++’ = positively associated, *p* < 0.001; ‘++’ = positively associated, *p* < 0.01; ‘+’ = positively associated, *p* < 0.05; ‘---’ = negatively associated, *p* < 0.001; ‘--’ = negatively associated, *p* < 0.01; ‘-’ = negatively associated, *p* < 0.05; ‘x’ not significantly associated). Details are shown in Additional file 1: Table S1

The uveitis activity parameters such as the ophthalmologist’s global assessment of uveitis activity, number of AC cells and the AC cell grade were positively associated among themselves (Fig. 2 and Additional file 1: Table S1). The VAS measures all correlated, the strongest was between the rheumatologist and patients’ assessment and the weakest between the ophthalmologist and patients’ assessment. The ophthalmologist VAS was most associated with AC activity and structural complications. It was more associated with visual acuity than the rheumatologist VAS. The rheumatologist VAS was most associated with the joint score as well as the patient-reported outcome measures, especially the CHAQ score. The patients’ VAS was most associated with ocular damage as well as the patients’ functional ability and quality of life measures.

#### Consensus about meausures for uveitis activity and damage

The definition of a response and damage index as well as the definition of uveitis remission and inactive disease was part of the second part of the meeting. The consensus process was based on the results of the prospectively followed uveitis patients within this study as reported in the first part of the results section.

The following items were selected for defining response to treatment

- Slit lamp evaluation:
  - ○ Total number of anterior chamber cells (AC)
  - ○ AC cells before pupil dilatation
- Grade of AC flare according to SUN criteria
- New Occurrence and course of inflammation-related structural complications:
  - ○ Occurrence of new posterior synechia
  - ○ Change of optic disc edema
  - ○ Change of macular edema
  - ○ Change of vitreous haze
- Ophthalmologist’s global assessment of uveitis activity in the worst eye assessed on a VAS score ranging between 0 to 100 mm
- Paediatric rheumatologist’s global assessment of disease activity assessed on a VAS score ranging between 0 to 100 mm
- Parents' (for patients < 8 years)/ patients rating about any problems with the eye assessed on a VAS score ranging between 0 to 100 mm
- Parents' (for patients < 8 years)/ patients rating about the course of uveitis during the last 3 months on a Likert scale (better / something better/ stable/ something worst/ worst)
- Parents (for patient’s age of under 8 years)/ patients rating about improvement under uveitis treatment during the last 3 months assessed on a VAS score ranging between 0 to 100 mm
- Change in health related quality of life, e.g. by the PedsQL, EYEQL …
- Missed work/school /kindergarten days due of the uveitis

Remission in JIAU on medication or off medication, analogous to the Wallace criteria for JIA [24]**,** is defined by fulfilling the following conditions for at least 6 months on medication, or inactive disease for > 3 months after discontinuation of all anti-inflammatory treatments for uveitis, respectively. In addition, inactive uveitis is defined by fulfilling the following conditions at a specific assessment. It is required that both eyes fulfil the following conditions

- Slit lamp total number of AC cells: 0 inflammatory cells. In aphakia patients some cells may be present in the anterior vitreous
- Absence of optic disc oedema
- Absence of macular oedema
- Absence of vitreous haze (< 0.5 +)
- Ophthalmologist global assessment of uveitis activity on VAS score ranging between 0 to 100 mm must be 0

The following items assess uveitis-related damage either in the right or left eye. The following complications define damage: visual acuity, synechiae, cataract, maculopathy, optic disc edema, decreased visual acuity, ocular hypertension (> 21 mmHg), ocular hypotony (< 6 mmHg), glaucomatous field loss and /or glaucomatous optic atrophy, band keratopathy, epiretinal membrane formation. Additionally, uveitis-related decreased visual acuity, visual deterioration of less than 0.3 in any eye, uveitis related disability reported by the ophthalmologist measured on a VAS ranging between 0 to 100 mm, uveitis related disability reported by the paediatric rheumatologist measured on a VAS ranging between 0 to 100 mm.

## Discussion

This study reports multiple outcomes of a representative population of patients with JIAU in a 6-month period following clinically-driven initiation of new DMARD treatment. The main findings are that the most significant changes, from baseline to 6 months, are found in the AC activity measures of cells and flare. These measures correlate with the presence of pre-existing structural complications and this has implications for the reporting of trials using a single measure as a primary outcome.

We also found that visual analogue scales of activity showed significant change when reported by the ophthalmologist, rheumatologist and families. The measures formed three relatively distinct groups. The first comprised uveitis activity and ocular damage and the ophthalmologists’ VAS. The second comprised patient-reported outcomes which included disruption to school attendance. The third consisted of the rheumatologists’ VAS, joint score and patient-reported outcomes.

There have been recent trials of adalimumab in JIAU using AC flare and an increase in AC cells as primary endpoints as well as a health economic analysis using visual acuity and surgical interventions as markers of health utility [6, 25, 26]. The variety of endpoints used in adult non-anterior uveitis, and the problems that arise from non-compatible studies has already been remarked upon [27, 28] and the same problem exists with chronic anterior uveitis in childhood [12]. In this study we confirm that AC cells are the most sensitive measure to change with onset of treatment that, with present knowledge, clinicians believe to be of most benefit in reducing the patients’ loss of function and quality of life caused by the disease. It also suggests that the three areas of AC cells and flare, patient reported functioning and quality of life and joint assessment should provide relatively separate areas of assessing the benefits of new treatments.

Although the primary aim of treatments is to reduce the rate of irreversible visual loss, this study emphasises the relatively minor value that changes in visual acuity have in the short-term assessment of change with treatment.

Standardized outcome measures of the lifelong outcome of JIAU are crucial. The SUN criteria [16] was used in the first clinical trial in paediatric uveitis, which led to the approval of a adalimumab for JIA associated uveitis [6], but formally it has not been validated. In addition few studies measure the impact of uveitis from a patient and parents perspective, as there is only one paediatric uveitis-specific quality of life assessment [18] for US-English speaking patients. A further deficit is the lack of disease specific measures of visual function, and generic measures of visual function used in trials of adults with macular disease or glaucoma may not adequately capture the range of visual disturbance found in JIAU including the high rates of amblyopia which create a disparity between ocular damage and patients-reported visual symptoms and function. Adults with good binocular vision prior to visual damage will have a much lower threshold for noticing visual symptoms and will suffer more from loss of binocular macular function than children with amblyopia developing early in the disease process.

Our group proposed a Paediatric Uveitis Outcome Measure (PROMS) in 2012 [1] in the present paper we have started validation by providing empirical evidence of those measures sensitive to change and how PROMS relate to indicators thought clinically relevant as prognosticators of lifelong disability. The importance of including patient and parent perspectives to capture the overall impact of disease [17], as well as the physicians’ global assessment of the disease activity has been shown by the significant size of these changes over time, and their relative independence from other metrics.

The introduction of the routine use of the ophthalmologist’s clinical judgment as a gold standard, through a visual analogue score for addressing inflammatory activity and severity of uveitis has problems. However, the strong correlation between the ophthalmologist global assessment and several clinical and instrumental variables (i.e. arthritis activity, laser flare photometry values) seems to assess the need to consider including the ophthalmologist’s perspective. This is now routine in several paediatric rheumatologic diseases where the rheumatologist’s global activity score is a recognised part of the activity assessment [29, 30]. The correlation of arthritis activity with the ophthalmologic global assessment is of interest as uveitis activity from clinical observation, does not seem to correlate with arthritis activity. The activity of joint disease will obviously influence the nature of the ophthalmic consultation and influence decisions about systemic management.

We have demonstrated multiple measures that demonstrate sensitivity to change over a clinically relevant period of time. Recent efficacy and health economic and drug efficacy studies have used additional measures and different time scales – it might be helpful to construct a single measure for all purposes such as the JADAS **(***Juvenile Arthritis Disease Activity Score)* scoring systems in JIA. A construct of “uveitis inflammatory activity” “UVEDAI”, for adult uveitis has been proposed, and some items used in this study are identical such as anterior chamber cell grade, vitreous haze, macula edema and patient-reported evaluations [31]. It does not include severity and damage items or a patient/parent global assessment. A comprehensive disease measure that is able to assess, at the same time and over the time, the overall picture of uveitis, combining both severity as well as activity, is still lacking.

Our finding that the primary measures of activity are mediated by coexisting damage confirm the clinical feeling that drug efficacy is unlikely to be the same in all eyes and therefore stratification by disease damage is likely to be necessary to obtain the best measure of drug efficacy. Although there are some situations where simplification such as a single, numeric disease score, is useful, there are other clinical situations where the complexity of the clinical course of JIAU is unavoidable and multiple measures will always be needed for an individualised assessment Evaluation of their relative weighting and redundancy is now required to minimise data collection for future targeted treatments.

We have also proposed definitions for treatment efficacy; remission, inactive disease and damage for JIAU. What remains most difficult to determine is the level and length of remission that is adequate to predict the two major outcomes of relevance to patients: the time of lifelong remission, and the elimination of risk of future visual loss. We intend to validate these outcomes in the frame of the MIWGUC working group.

Our study had some limitations. The patient reported outcomes were not available for all patients at enrolment and in follow-up due to administrative reasons. Therefore, the analyses may lack of statistical power. In addition, there were a considerable number of patients lost to follow-up in our study. However, we did not find that the missing data biased the results. We also did not include patient or carer participation at this stage of the process.

This work is the initial step for developing a comprehensive assessment of outcomes of children with JIAU which incorporates the perspectives of rheumatologists, ophthalmologists, patients and families. We propose measures of disease activity, severity and damage. A standardized method to assess the clinical characteristics of the disease will also serve as a useful tool to compare JIAU patients children within and between clinical trials.

## Conclusion

We propose measures of disease activity, severity and damage based on our prospective validation study of our previously proposed outcome measures [14]. This work is the initial step for developing a comprehensive outcome measures for JIAU, that incorporate the perspectives of rheumatologists, ophthalmologists, patients and families.

## Additional file

Additional file 1: **Table S1.** Associations parameters. Associations between parameters that are associated to JIA and uveitis and patient reported outcomes (bold highlighted beta/ OR were statistically significant). (DOCX 30 kb)

## Data Availability

Please contact author for data requests.

## Abbreviations

AC: Anterior chamber
ANA: Antinuclear antibodies
bDMARDS: Biologic modifier drugs
CHAQ: Childhood Health Assessment Questionnaire
CI: Confidence interval
COMET: Core Outcome Measures in Effectiveness Trials
csDMARD: Conventional synthetic disease-modifying anti-rheumatic drug
DMARDS: Disease-modifying antirheumatic drugs
HLA: Human leukocyte antigen
ILAR: International League of Associations for Rheumatology
IOP: Intraocular pressure
JADAS: *Juvenile Arthritis Disease Activity Score*
JIA: Juvenile Idiopathic Arthritis
JIAU: JIA-associated uveitis
logMAR: Logarithm of the minimum angle of resolution
MIWGUC: Multinational Interdisciplinary Working Group for Uveitis in Childhood
n: Number of patients with valid assessment in the reported parameter
NSAIDs: Non-steroidal anti-inflammatory drugs
OCT: Optical coherence tomography
OMERACT: Outcome Measures in Rheumatology
OR: Odds ratio for categorical variables
PedsQL: Paediatric Quality of Life Inventory
PROMS: Proposed outcome measures
*p*-value: Probability value
RCT: Randomized controlled trial
RF: Rheumatoid factor
SD: Standard deviation
SUN: Standardization of Uveitis Nomenclature
TNF: Tumor necrosis factor
UVEDAI: Uveitis inflammatory activity
VAS: Visual analogue scale range 0 to 100
