# Correction to: Proposal for a definition for response to treatment, inactive disease and damage for JIA associated uveitis based on the validation of a uveitis related JIA outcome measures from the Multinational Interdisciplinary Working Group for Uveitis in Childhood (MIWGUC)

**DOI:** 10.1186/s12969-019-0396-4

**Published:** 2020-02-11

**Authors:** Ivan Foeldvari, Jens Klotsche, Gabriele Simonini, Clive Edelsten, Sheila T. Angeles-Han, Regitze Bangsgaard, Joke de Boer, Gabriele Brumm, Rosa Bou Torrent, Tamas Constantin, Cinzia DeLibero, Jesus Diaz, Valeria Maria Gerloni, Margarida Guedes, Arnd Heiligenhaus, Kaisu Kotaniemi, Sanna Leinonen, Kirsten Minden, Vasco Miranda, Elisabetta Miserocchi, Susan Nielsen, Martina Niewerth, Irene Pontikaki, Carmen Garcia de Vicuna, Carla Zilhao, Steven Yeh, Jordi Anton

**Affiliations:** 1 Head of the Hamburg Centre for Pediatric and Adolescence Rheumatology Centre for Treatment of Scleroderma and Uveitis in Childhood and Adolescence Teaching Unit of the Asklepios Campus of the Semmelweis Medical School, Budapest An der Schön Klinik Hamburg Eilbek Dehnhaide, 120 22081 Hamburg, Germany; 2 German Rheumatism Research Centre, 10117 Berlin, Germany; 3 Institute for Social Medicine, Epidemiology, and Health Economics, Charité Universitaetsmedizin Berlin, Berlin, Germany; 4 Rheumatology Unit- A. Meyer Children’s Hospital- NEUROFARBA Department, University of Florence, Florence, Italy; 5 Department Rheumatology, Great Ormond Street Hospital, Great Ormond Street, London, UK; 6 Division of Rheumatology, Cincinnati Children’s Hospital Medical Center, 3333 Burnett; Avenue, Cincinnati, OH 45229; Department of Pediatrics, University of Cincinnati, Cincinnati, OH USA; 7 Department of Ophthalmology, Copenhagen University Hospital Glostrup/Rigshospitalet, Copenhagen, Denmark; 8 UMC Utrecht, Utrecht, Netherlands; 9 Universitätsklinikum Hamburg-Eppendorf, Hamburg, Germany; 10 Pediatric Rheumatology Department, Hospital Sant Joan de Déu, Barcelona, Spain; 11 2nd Department of Pediatrics, Semmelweis University, Budapest, Hungary; 12 AOU Meyer, Florence, Italy; 13 Università di Milano - Istituto Gaetano Pini, Milan, Italy; 14 Pediatric Rheumatology Unit, Centro Hospitalar Universitário do Porto, Porto, Portugal; 15 St. Franziskus-Hospital Münster, Muenster, Germany; 16 Department of Ophthalmology, Helsinki University Hospital, Helsinki, Finland; 17 Charité – Universitätsmedizin Berlin, corporate member of Freie Universität Berlin, Humboldt-Universität zu Berlin, and Berlin Institute of Health; Department of Rheumatology and Clinical Immunology, Berlin, Germany; 18 Pediatric Ophthalmologist at the Centro Hospitalar Universitario do Porto, Teaching Unit of the Abel Salazar Institute of Biomedical Sciences, Largo do Prof. Abel Salazar, 4099-001 Porto, Portugal; 19 Ocular Immunology and Uveitis Service, Department of Ophthalmology, San Raffaele Scientific, Institute, Via Olgettina 60, 20122 Milan, Italy; 20 Emory Clinic, Atlanta, USA; 21 Institut de Recerca Sant Joan de Déu, Barcelona, Spain; 22 Department of Surgery and Surgery Specializations, Universitat de Barcelona, Barcelona, Spain; 23 Ophtalmology Department, Hospital Sant Pau, Barcelona, Spain; 24 Ophtalmology Department, Hospital Sant Joan de Déu, Barcelona, Spain

**Correction to: Pediatr Rheumatol Online J (2019) 17:66**

**https://doi.org/10.1186/s12969-019-0345-2**

Following publication of the original article [1], we have been notified that the author Joan Calzada should not have been included to the team of authors. The authors’ team, thus, should be as follows:

Ivan Foeldvari1*†, Jens Klotsche2,3†, Gabriele Simonini4, Clive Edelsten5, Sheila T. Angeles-Han6, Regitze Bangsgaard7,Joke de Boer8, Gabriele Brumm9, Rosa Bou Torrent10,21, Tamas Constantin11, Cinzia DeLibero12, Jesus Diaz23,24,Valeria Maria Gerloni13, Margarida Guedes14, Arnd Heiligenhaus15, Kaisu Kotaniemi16, Sanna Leinonen16,Kirsten Minden2,17, Vasco Miranda18, Elisabetta Miserocchi19, Susan Nielsen7, Martina Niewerth2, Irene Pontikaki13,Carmen Garcia de Vicuna10, Carla Zilhao14, Steven Yeh20, Jordi Anton10,21,22
