# Supplementary material for: Proposal for a definition for response to treatment, inactive disease and damage for JIA associated uveitis based on the validation of a uveitis related JIA outcome measures from the Multinational Interdisciplinary Working Group for Uveitis in Childhood (MIWGUC)
Source: Pediatr Rheumatol Online J. 2019 Oct 1;17:66. doi: 10.1186/s12969-019-0345-2 (PMC6774210; doi:10.1186/s12969-019-0345-2)
Supplement: Supplementary file 1 — Table S1. Associations parameters. Associations between parameters that are associated to JIA and uveitis and patient reported outcomes (bold highlighted beta/ OR were statistically significant). (DOCX 30 kb) [file 12969_2019_345_MOESM1_ESM.docx]

Additional file 1: Table S1: Associations parameters

|  |  |  |  |  |  |  |  |  |  |  |  |  |  |  |  |  |  |  |  |  |
| --- | --- | --- | --- | --- | --- | --- | --- | --- | --- | --- | --- | --- | --- | --- | --- | --- | --- | --- | --- | --- |
|  | ***Ophthalmologist*** | | | | | | | | | |  | ***Pediatric rheumatologist*** | |  | ***Patients reported outcomes*** | | | | | |
|  |  |  |  |  |  |  |  |  |  |  |  |  |  |  |  |  |  |  |  |  |
|  | **Physician's global about disease activity in the eyes, VAS, 0-100** | **Total number of AC cells** | **AC cell grade** |  | **Visual acuity, logMAR** |  | **AC flare grade (flare versus no flare), SUN** | **AC flare grade (flare versus no flare), MIWGUC** |  | **Any structural complication** |  | **Physician's global about JIA disease activity, VAS, 0-100** | **Number of joints with active arthritis** |  | **Overall well-being; VAS; 0-100** | **C-HAQ, 0-3** | **PedsQL, 0-100** | **Number of days in hospital due to uveitis** | **Missed days in kindergarden/ school due to uveitis** | **Number of days with restrictions in daily life due to uveitis** |
|  |  |  |  |  |  |  |  |  |  |  |  |  |  |  |  |  |  |  |  |  |
|  | **beta/OR 95%CI p value** | **beta/OR 95%CI p value** | **beta/OR 95%CI p value** |  | **beta/OR 95%CI p value** |  | **beta/OR 95%CI p value** | **beta/OR 95%CI p value** |  | **beta/OR 95%CI p value** |  | **beta/OR 95%CI p value** | **beta/OR 95%CI p value** |  | **beta/OR 95%CI p value** | **beta/OR 95%CI p value** | **beta/OR 95%CI p value** | **beta/OR 95%CI p value** | **beta/OR 95%CI p value** | **beta/OR 95%CI p value** |
|  |  |  |  |  |  |  |  |  |  |  |  |  |  |  |  |  |  |  |  |  |
| ***Pediatric rheumatologist*** | | |  |  |  |  |  |  |  |  |  |  |  |  |  |  |  |  |  |  |
| Physician's global about JIA disease activity, VAS, 0-100 | **0.35 <0.001 0.26 ; 0.45** | **0.80 <0.001 0.37 ; 1.23** | **4.36 <0.001 2.10 ; 6.61** |  | **8.88 0.009 2.18 ; 15.57** |  | 4.54 0.125 -1.27 ; 10.34 | -3.56 0.356 -11.11 ; 3.99 |  | 5.55 0.052 -0.04 ; 11.15 |  | - | **2.22 <0.001 1.21 ; 3.22** |  | **0.58 <0.001 0.47 ; 0.69** | **12.34 <0.001 8.31 ; 16.38** | **-0.60 <0.001 -0.75 ; -0.45** | **1.54 <0.001 0.86 ; 2.21** | **1.23 <0.001 0.90 ; 1.57** | 0.29 0.072 -0.03 ; 0.60 |
| Number of joints with active arthritis | -0.01 0.159 -0.02 ; 0.00 | 0.02 0.529 -0.04 ; 0.08 | -0.04 0.684 -0.26 ; 0.17 |  | -0.39 0.234 -1.02 ; 0.25 |  | -0.35 0.191 -0.87 ; 0.17 | -0.56 0.131 -1.29 ; 0.17 |  | -0.47 0.084 -1.00 ; 0.06 |  | **0.02 <0.001 0.01 ; 0.03** | - |  | **0.02 0.021 0.00 ; 0.04** | -0.03 0.928 -0.70 ; 0.64 | **-0.04 <0.001 -0.06 ; -0.02** | **0.17 0.001 0.07 ; 0.28** | -0.01 0.824 -0.06 ; 0.04 | **0.07 0.003 0.02 ; 0.12** |
|  |  |  |  |  |  |  |  |  |  |  |  |  |  |  |  |  |  |  |  |  |
| ***Patients reported outcomes*** | | |  |  |  |  |  |  |  |  |  |  |  |  |  |  |  |  |  |  |
| Overall well-being; VAS; 0-100 | **0.22 <0.001 0.11 ; 0.33** | -0.44 0.122 -0.99 ; 0.12 | **4.22 0.001 1.82 ; 6.63** |  | -1.12 0.786 -9.25 ; 7.00 |  | 6.65 0.057 -0.20 ; 13.49 | 0.32 0.943 -8.39 ; 9.02 |  | 0.92 0.813 -6.75 ; 8.59 |  | **0.61 <0.001 0.51 ; 0.72** | **1.23 0.023 0.17 ; 2.28** |  | - | **11.26 <0.001 6.26 ; 16.26** | **-0.71 <0.001 -0.89 ; -0.53** | **1.34 0.001 0.54 ; 2.14** | **0.94 <0.001 0.54 ; 1.35** | **0.39 0.037 0.02 ; 0.76** |
| C-HAQ, 0-3 | 0.00 0.908 0.00 ; 0.01 | 0.01 0.815 -0.06 ; 0.07 | -0.05 0.429 -0.17 ; 0.07 |  | **-0.40 0.009 -0.70 ; -0.10** |  | **-0.59 <0.001 -0.93 ; -0.26** | **-0.69 <0.001 -1.03 ; -0.36** |  | **-0.66 <0.001 -1.04 ; -0.29** |  | **0.02 <0.001 0.01 ; 0.02** | -0.01 0.818 -0.05 ; 0.04 |  | **0.01 <0.001 0.01 ; 0.02** | - | **-0.02 <0.001 -0.03 ; -0.02** | 0.02 0.149 -0.01 ; 0.05 | 0.01 0.155 -0.01 ; 0.03 | **0.01 0.032 0.00 ; 0.03** |
| PedsQL, 0-100 | -0.04 0.356 -0.14 ; 0.05 | 0.10 0.712 -0.42 ; 0.61 | -0.36 0.752 -2.56 ; 1.85 |  | 5.53 0.055 -0.12 ; 11.17 |  | **7.10 0.010 1.70 ; 12.51** | **7.00 0.019 1.15 ; 12.85** |  | 4.38 0.126 -1.23 ; 9.99 |  | **-0.41 <0.001 -0.51 ; -0.31** | **-1.17 0.002 -1.92 ; -0.42** |  | **-0.49 <0.001 -0.62 ; -0.37** | **-12.20 <0.001 -15.40 ; -8.99** | - | **-1.56 <0.001 -2.11 ; -1.00** | **-0.74 <0.001 -1.09 ; -0.38** | -0.25 0.052 -0.51 ; 0.00 |
| Number of days in hospital due to uveitis | -0.01 0.674 -0.03 ; 0.02 | 0.00 0.556 -0.01 ; 0.01 | 0.59 0.088 -0.09 ; 1.27 |  | -0.01 0.993 -1.67 ; 1.66 |  | -1.49 0.083 -3.18 ; 0.19 | -1.65 0.058 -3.36 ; 0.05 |  | 0.28 0.764 -1.55 ; 2.11 |  | **0.06 <0.001 0.03 ; 0.09** | **0.35 0.002 0.13 ; 0.57** |  | **0.07 <0.001 0.03 ; 0.11** | 0.80 0.149 -0.29 ; 1.88 | **-0.11 <0.001 -0.15 ; -0.07** | - | **0.23 <0.001 0.18 ; 0.29** | **0.08 0.001 0.03 ; 0.12** |
| Missed days in kindergarden/ school due to uveitis | **0.06 0.001 0.02 ; 0.09** | -0.05 0.477 -0.17 ; 0.08 | 0.80 0.052 -0.01 ; 1.61 |  | -0.48 0.649 -2.53 ; 1.58 |  | 0.61 0.571 -1.49 ; 2.70 | 0.27 0.819 -2.05 ; 2.59 |  | 1.75 0.119 -0.45 ; 3.94 |  | **0.15 <0.001 0.11 ; 0.19** | -0.03 0.866 -0.39 ; 0.33 |  | **0.12 <0.001 0.08 ; 0.17** | 1.18 0.155 -0.45 ; 2.82 | **-0.11 <0.001 -0.17 ; -0.05** | **1.30 <0.001 0.98 ; 1.62** | - | 0.06 0.242 -0.04 ; 0.17 |
| Number of days with restrictions in daily life due to uveitis | -0.02 0.488 -0.08 ; 0.04 | -0.14 0.338 -0.43 ; 0.15 | 0.10 0.892 -1.39 ; 1.60 |  | 0.44 0.809 -3.15 ; 4.04 |  | 2.62 0.161 -1.04 ; 6.29 | 0.50 0.795 -3.24 ; 4.23 |  | -1.30 0.523 -5.28 ; 2.69 |  | 0.06 0.126 -0.02 ; 0.13 | **0.74 0.005 0.22 ; 1.26** |  | **0.11 0.021 0.02 ; 0.20** | **2.59 0.032 0.22 ; 4.97** | **-0.12 0.013 -0.21 ; -0.03** | **0.90 0.001 0.37 ; 1.43** | 0.13 0.262 -0.10 ; 0.36 | - |
|  |  |  |  |  |  |  |  |  |  |  |  |  |  |  |  |  |  |  |  |  |
| ***Ophthalmologist*** | | |  |  |  |  |  |  |  |  |  |  |  |  |  |  |  |  |  |  |
| Physician's global about disease activity in the eyes, VAS, 0-100 | - | 0.21 0.473 -0.37 ; 0.79 | **10.01 <0.001 7.49 ; 12.54** |  | **23.25 <0.001 15.46 ; 31.04** |  | **17.84 <0.001 11.17 ; 24.52** | **16.74 <0.001 8.50 ; 24.99** |  | **22.77 <0.001 16.02 ; 29.53** |  | **0.51 <0.001 0.40 ; 0.63** | -1.20 0.067 -2.49 ; 0.08 |  | **0.21 0.012 0.05 ; 0.37** | -1.85 0.529 -7.61 ; 3.91 | 0.02 0.842 -0.21 ; 0.26 | -0.74 0.098 -1.63 ; 0.14 | **0.72 0.004 0.23 ; 1.21** | -0.33 0.126 -0.74 ; 0.09 |
| Total number of AC cells | 0.07 0.106 -0.01 ; 0.16 | - | **4.91 <0.001 4.15 ; 5.67** |  | **4.79 <0.001 2.26 ; 7.32** |  | **4.44 <0.001 2.37 ; 6.51** | **4.29 0.001 1.81 ; 6.76** |  | 0.63 0.502 -1.22 ; 2.48 |  | **0.17 <0.001 0.09 ; 0.25** | 0.23 0.379 -0.28 ; 0.75 |  | -0.08 0.216 -0.21 ; 0.05 | 1.49 0.084 -0.20 ; 3.17 | 0.05 0.218 -0.03 ; 0.14 | -0.74 0.827 -7.36 ; 5.88 | -0.13 0.608 -0.61 ; 0.36 | -0.04 0.692 -0.26 ; 0.17 |
| AC cell grade | **0.02 <0.001 0.01 ; 0.02** | **0.13 <0.001 0.11 ; 0.15** | - |  | 0.01 0.991 -0.30 ; 0.30 |  | **1.29 <0.001 1.07 ; 1.52** | **1.14 <0.001 0.85 ; 1.43** |  | **0.46 <0.001 0.21 ; 0.72** |  | **0.01 0.009 0.00 ; 0.01** | -0.01 0.697 -0.05 ; 0.04 |  | **0.01 0.017 0.00 ; 0.02** | -0.16 0.184 -0.40 ; 0.08 | 0.00 0.709 -0.01 ; 0.01 | 0.03 0.196 -0.01 ; 0.07 | 0.02 0.104 0.00 ; 0.04 | 0.00 0.978 -0.02 ; 0.02 |
| Visual acuity, logMAR | **0.01 <0.001 0.00 ; 0.01** | 0.01 0.716 -0.01 ; 0.01 | -0.01 0.571 -0.05 ; 0.03 |  | - |  | **0.11 0.028 0.01 ; 0.20** | 0.06 0.339 -0.06 ; 0.17 |  | **0.18 0.001 0.07 ; 0.28** |  | **0.01 0.014 0.00 ; 0.00** | -0.01 0.156 -0.03 ; 0.00 |  | 0.01 0.614 0.00 ; 0.00 | **-0.13 0.008 -0.22 ; -0.03** | 0.01 0.071 0.00 ; 0.01 | 0.01 0.819 -0.02 ; 0.01 | 0.01 0.978 -0.01 ; 0.01 | 0.01 0.813 -0.01 ; 0.01 |
| AC flare grade (flare versus no flare), SUN | **1.03 <0.001 1.01 ; 1.04** | **1.25 <0.001 1.10 ; 1.41** | **3.76 <0.001 2.81 ; 5.04** |  | **2.36 0.010 1.23 ; 4.55** |  | - | - |  | **4.52 <0.001 2.36 ; 8.69** |  | 1.01 0.372 0.99 ; 1.02 | 0.92 0.179 0.80 ; 1.04 |  | 1.01 0.341 0.99 ; 1.02 | **0.49 0.002 0.32 ; 0.77** | **1.02 0.015 1.00 ; 1.05** | 0.93 0.085 0.86 ; 1.01 | 1.01 0.773 0.96 ; 1.05 | 1.03 0.210 0.99 ; 1.07 |
| AC flare grade (flare versus no flare), MIWGUC | **1.02 <0.001 1.01 ; 1.03** | **1.25 0.004 1.07 ; 1.46** | **2.89 <0.001 2.10 ; 3.99** |  | 1.43 0.276 0.75 ; 2.71 |  | - | - |  | **4.68 <0.001 2.19 ; 10.00** |  | 0.99 0.289 0.98 ; 1.01 | 0.86 0.156 0.70 ; 1.06 |  | 1.00 0.686 0.99 ; 1.02 | **0.45 0.001 0.29 ; 0.71** | **1.02 0.045 1.00 ; 1.04** | 0.92 0.081 0.84 ; 1.01 | 1.00 0.995 0.96 ; 1.05 | 1.00 0.917 0.97 ; 1.04 |
| Any structural complication | **1.03 <0.001 1.02 ; 1.04** | 0.99 0.869 0.91 ; 1.08 | **1.38 0.004 1.11 ; 1.71** |  | **4.58 0.004 1.64 ; 12.76** |  | **3.19 <0.001 1.84 ; 5.54** | **3.83 <0.001 1.92 ; 7.63** |  | - |  | 1.01 0.085 1.00 ; 1.02 | 0.91 0.065 0.83 ; 1.01 |  | 1.00 0.949 0.98 ; 1.02 | **0.47 0.002 0.30 ; 0.75** | 1.01 0.131 1.00 ; 1.03 | 1.01 0.820 0.92 ; 1.11 | 1.08 0.066 1.00 ; 1.17 | 0.99 0.462 0.95 ; 1.02 |
|  |  |  |  |  |  |  |  |  |  |  |  |  |  |  |  |  |  |  |  |  |
| beta = regression coefficient for continuously distributed variables; CI = confidence interval; OR = Odds ratio for categorical variables | | | | | | | | | | | | | | | | | |  |  |  |
